# Cyclodextrin and KR12-Lipopeptide Interactions: A Thermodynamic View of Binding Mechanisms and Impact on the Structure of α‑Helical Peptides

**DOI:** 10.1021/acs.jpcb.5c06749

**Published:** 2026-01-19

**Authors:** Martyna Kapica, Ola Grabowska, Elżbieta Kamysz, Julia Kamysz, Sergey A. Samsonov, Dariusz Wyrzykowski

**Affiliations:** † Faculty of Chemistry, University of Gdańsk, Wita Stwosza 63, 80-308 Gdańsk, Poland; ‡ Inter-faculty Individual Studies in Mathematics and Natural Sciences, University of Warsaw, Stefana Banacha 2C, 02-097 Warsaw, Poland

## Abstract

Natural antimicrobial peptides are a promising new class of antibiotics that offer a potential response to the growing challenge of microbial resistance. Among these, lipopeptides—cationic peptides conjugated with fatty acid residues—are especially noteworthy as their tendency to form α-helical structures plays a crucial role in determining their biological effectiveness. The ability to adopt an ordered structure, which directly influences their antimicrobial action, is determined by the length of the conjugated alkyl chain. In this study, experimental methods (isothermal titration calorimetry and circular dichroism), complemented by in silico analysis, have been successfully applied to rigorously characterize the interactions between three types of cyclodextrin (α-, ß-, and γ-) and three variants of antimicrobial KR12-lipopeptides, namely, the octanoylated (C8-KR12-NH_2_), laurylated (C12-KR12-NH_2_), and myristoylated (C14-KR12-NH_2_) KR12-lipopeptide. This integrated approach allowed, for the first time, the extraction of detailed structural and dynamic insights into the mechanism by which KR12 guests insert into the cyclodextrin cavity host. Furthermore, it was proven that encapsulation of the hydrophobic chain within the cyclodextrin cavity disrupts intramolecular interactions stabilizing the α-helical structure, leading to a reduced content of α-helicity. These findings underscore the role of cyclodextrins as modulators of the structural and functional properties of α-helical lipopeptides.

## Graphical abstract

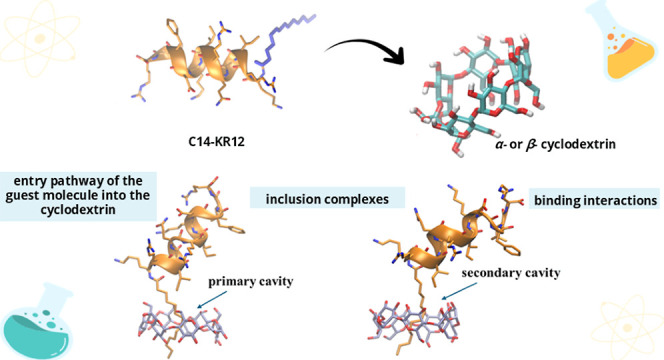

## 1 Introduction

Natural antimicrobial peptides (AMPs) and their synthetic derivatives are the subject of extensive ongoing research as they show great potential as a new class of antibiotics for combating antimicrobial resistance.^1^ The variety of AMPs, in terms of their mechanisms of action and modes of administration, allows for their evaluation across a wide range of clinical applications.^2^ Among the most promising AMPs, particular attention has been paid to lipopeptides, i.e., cationic antibacterial peptides conjugated with fatty acid residues. Incorporating hydrophobic moieties into the peptide structure enhances antimicrobial efficacy against both Gram-negative and Gram-positive bacteria.^3,4^ The amino acid sequence enables bacterial recognition through long-range electrostatic interactions and is crucial for targeting Gram-negative bacteria, while the hydrophobic fatty acid chain assists in anchoring the peptide to bacterial membranes via van der Waals forces, leading to membrane disruption.

The special subclass of AMPs comprises α-helical peptides that tend to adopt an α-helical conformation in response to specific environmental conditions or in contact with bacterial membranes. α-Helices can be involved in interactions between proteins, nucleic acids, and the lipids of cell membranes. The α-helical structure of AMPs is affected by various physicochemical factors, such as temperature, pH, ionic strength, the presence of metal ions, and membrane-like environments. In addition, certain structural features of this group of peptides—such as amino acid composition, side-chain interactions, and capping motifs—also play a crucial role in promoting the α-helical conformation.^5^ Therefore, the conformation of the lipopeptide is determined by the synergistic effect of environmental conditions and its structural features, which can significantly alter its interaction with bacterial membranes.

In the case of lipopeptides, the length of the hydrophobic alkyl chain plays a significant role in determining their capacity to adopt this particular secondary structure. Such behavior has been observed for a series of lipopeptides representing the conjugation of the KR12 peptide (K18R19IVQR23IK25DFLR29-NH2), the smallest AMP derived from human cathelicidin LL-37, with some fatty acid residues.^4^ It was demonstrated that under the same experimental conditions, the KR12-lipopeptides with longer *n*-alkyl chains easily obtain the desirable α-helical conformation, which enhances their strong antimicrobial effects when adopting an α-helix structure.^4,6,7^ Incorporating a longer fatty acid chain into the structure of AMPs increases their lipophilicity. However, optimal hydrophobicity is essential for maximum activity since excessively high or low lipophilicity can reduce the antimicrobial effectiveness of the peptide.^8,9^

Recently, we have reported the physicochemical nature of cyclodextrin (circular dichroism (CD)) interactions with some low-molecular-weight ligands comprising different alkyl chain lengths.^10,11^ The formation of fairly stable host–guest complexes was considered in terms of the different entrance modes of the ligand (tail vs head) into the cyclodextrin cavity (primary vs secondary), depending on the length of the hydrophobic moiety of the ligand. Based on previous findings, this paper presents the results aimed at assessing the potential of cyclodextrins as guest–host complex-forming agents with lipopeptides. Particular attention was paid to the analysis of the physicochemical mechanisms driving the cyclodextrin–lipopeptide interactions and their impact on the secondary structure of α-helical lipopeptides.

It is reasonably hypothesized that the interaction between cyclodextrins and the hydrophobic moiety of the lipopeptide will disrupt the interactions of the fatty acid residue chain with the peptide backbone, which is responsible for stabilization of the α-helix structure. This disruption may occur as the hydrophobic chain becomes encapsulated within the cyclodextrin cavity, effectively trapping it. Consequently, the formation of host–guest complexes may lead to a decrease in the α-helix content of the lipopeptide, potentially influencing its structural and functional properties. Furthermore, it has also been proven that the formation of cyclodextrin–lipopeptide complexes prevents the formation of lipopeptide aggregates.^12^ This manifests itself in reduced toxicity to various human cells, while maintaining the antibacterial activity of lipopeptides. The presence of a hydrophobic fatty acid residue (the “tail”) promotes the formation of micelle-like assemblies, similar to those observed with surfactants or long-chain ionic liquids.^13^ Therefore, it can be tentatively assumed that the formation of peptide aggregates will facilitate the adoption of an α-helical conformation by the peptide segment (the “head”). To verify the hypothesis concerning the influence of cyclodextrin on the secondary structure of α-helical lipopeptides, we investigated the underlying physicochemical principles of these interactions. Specifically, we examined the interactions between three types of cyclodextrin (α-, ß- and γ-) and three variants of KR12-lipopeptides, namely, the octanoylated (C8-KR12-NH_2_), laurylated (C12-KR12-NH_2_), and myristoylated (C14-KR12-NH_2_) KR12-lipopeptide.

The relationships between the structure, physicochemical properties, and biological activity of peptides remain a significant and intriguing challenge in peptide engineering. Understanding the host and guest factors that influence the stability of cyclodextrin–lipopeptide complexes can help to optimize AMP formulations. This knowledge may also offer valuable insights in the future on strategies to enhance the efficacy, stability, and selective cytotoxicity of AMPs.^12,14^

## 2 Materials and Methods

### 2.1 Reagents

Sodium cacodylate trihydrate (Caco, ≥98%) was obtained from Merck (Warszawa, Poland). α-cyclodextrin (α-CD, ≥98%, CAS: 10016-20-3) and β-cyclodextrin (β-CD, ≥97%, CAS: 7585-39-9) were purchased from Sigma-Aldrich (Poland), γ-cyclodextrin (γ-CD, ≥98%, CAS: 17465-86-0) was purchased from Apollo Scientific (UK) and used without further purification. Double-distilled water with a conductivity not exceeding 0.18 μS cm^–1^ was used for preparations of buffer solutions.

### 2.2 Synthesis of the C8-KR12-NH_2_, C12-KR12-NH_2_, and C14-KR12-NH_2_ Peptides

The peptides C8-KR12-NH_2_ (octanoic acid-KRIVQRIKDFLR-NH_2_), C12-KR12-NH_2_ (dodecanoic acid-KRIVQRIKDFLR-NH_2_), and C14-KR12-NH_2_ (tetradecanoic acid-KRIVQRIKDFLR-NH_2_) were synthesized manually by the solid-phase method using 9-fluorenylmethoxycarbonyl (Fmoc) chemistry on resin modified by a Rink amide linker resin with loading of 1.0 mmol/g (Orpegen Peptide Chemicals GmbH, Heidelberg, Germany) according to the procedure described in our previous work.^4^ *N-*α-Fmoc-protected amino acids used for the solid-phase synthesis were obtained from Iris Biotech GmbH (Marktredwitz, Germany). The following amino acid derivatives were used: Fmoc-Lys­(Boc)-OH, Fmoc-Arg­(Pbf)-OH, Fmoc-Ile-OH, Fmoc-Val-OH, Fmoc-Gln­(Trt)-OH, Fmoc-Asp­(OtBu)-OH, Fmoc-Phe-OH, and Fmoc-Leu-OH.

All peptides were removed from the resin, along with side-chain deprotection, in a one-step procedure using a mixture of trifluoroacetic acid (TFA; Apollo Scientific, Denton, UK), triisopropylsilane (TIS; Sigma-Aldrich, St. Louise, MO, USA), and water (95:2.5:2.5 v/v/v)^4^. Finally, the peptides were purified by solid-phase extraction (SPE) on Isolute TM SPE columns (flash, C18, 25 mL).^15^

The purity of the peptides after purification was at least 95%, as determined by analytical reversed-phase high-performance liquid chromatography. Their identity was confirmed by electrospray ionization mass spectrometry.

### 2.3 Isothermal Titration Calorimetry

Isothermal titration calorimetry (ITC) studies were carried out using an Auto-ITC instrument (MicroCal, GE Healthcare, Northampton, USA). All reagents were prepared in double-distilled water. The sample cell was filled with a 0.2 mM solution of the lipopeptide CX-KR12-NH_2_, where X denotes an alkyl chain consisting of 14, 12, or 8 carbon atoms. The titrations were conducted using 4 mM solutions of either α-cyclodextrin (α-CD) or β-cyclodextrin (β-CD) as the titrant. Each injection lasted 20 s, with 240 s intervals between successive injections. Additionally, the heat of dilution of the titrant was determined in separate control experiments and subtracted from the cyclodextrin–lipopeptide titration data.

### 2.4 CD Spectroscopy

Spectral data were obtained using a Jasco-715 automated spectropolarimeter (Jasco Inc., USA). The tested solutions contained lipopeptide (C14-KR12-NH_2_ or C12-KR12-NH_2_) and a ligand (α-CD or β-CD) at molar ratios of 1:0 and 1:4. The lipopeptide concentration was 0.1 mM. All samples were prepared in 50 mM Caco buffer at pH 5. CD spectra were collected in 1 mm quartz cuvettes (sample volume: 0.3 mL) over the wavelength range of 190–260 nm, with a sensitivity of 5° and a scan speed of 50 nm min^–1^. Measurements were conducted at 298.15 K. Changes in the lipopeptide secondary structure were analyzed using the Dichroweb online server.^17^

### 2.5 Molecular Dynamics Simulations

The structures of host–guest complexes containing α-, β-, and γ-cyclodextrins (hosts) and C14-KR12-NH_2_, C12-KR12-NH_2_, or C8-KR12-NH_2_ (guests) were constructed using the Xleap module of the AMBER16 software package.^18^ The peptide fragment KR12 was obtained from the PDB (PDB ID: 2K6O, the first NMR model). The initial complexes were built by manually inserting an aliphatic tail of a guest molecule into the cyclodextrin cavity on both sides.

Molecular dynamics (MD) simulations were performed using the AMBER16 software package.^18^ Each complex was placed in a truncated octahedral periodic box filled with TIP3P water, maintaining a minimum distance of 15 Å between the solute and the box boundary. The overall system charge was neutralized with chloride (Cl^–^) counterions.

Parameters for both the cyclodextrin host were taken from GLYCAM06,^19^ while the guest molecules aliphatic tails (C14-, C12-, and C8-) were generated using the antechamber module of AMBER16,^18^ employing the GAFF force field^20^ together with RESP charges.^21^

The energy minimization procedure was performed in two stages. Initially, 500 cycles of the steepest descent algorithm were followed by 1000 cycles of the conjugate gradient method, both under harmonic positional restraints of 100 kcal/mol/Å^2^. This was followed by an additional 3000 cycles of steepest descent and 3000 cycles of conjugate gradient minimization, this time without any restraints. Upon completion of minimization, the system was gradually heated from 0 to 300 K over 10 ps under the same harmonic restraints. Equilibration was performed for 500 ps under constant temperature (300 K) and pressure (10^5^ Pa) conditions, ensuring the thermal and baric stability of the system. The production phase of the MD simulation was subsequently conducted for 100 ns in the same isothermal–isobaric ensemble, providing adequate sampling of the conformational landscape of the host–guest complexes. The particle mesh Ewald technique was utilized to accurately account for electrostatic interactions, while the SHAKE algorithm was employed to constrain all covalent bonds involving hydrogen atoms throughout the molecular dynamics simulations. Equilibration was considered achieved when the complex structure demonstrated stability, as indicated by the root-mean-square deviation (RMSD) values, which plateaued within a few nanoseconds.

#### 2.5.1 Binding Free-Energy Calculations

Energetic postprocessing, including per-residue energy decomposition, was carried out for all systems using the linear interaction energy (LIE) method with a dielectric constant of 80, together with Molecular Mechanics Generalized Born Surface Area (MM-GBSA) calculations.^22^ The MM-GBSA analyses employed the igb = 2 solvent model implemented in AMBER16,^18^ which uses default parameters for Born radii and solvent-accessible surface area.

#### 2.5.2 Umbrella Sampling

For each complex, the initial structure for the umbrella sampling (US) simulation was selected from the MD trajectory frame and displayed the minimum RMSD value relative to the average conformation of the guest molecule throughout the simulation. The distance between the C-end (the first carbon atom C1 of the hydrocarbon chain of the lipopeptide, which was covalently bound to the peptide) and the center of mass of each cyclodextrin (α-, β-, and γ-CD) was measured and used as the reaction coordinate. The bound conformation was used as the initial structure, and the peptide–guest molecule was separated gradually along the defined reaction coordinate with a step size of 1.0 Å for 40 Å. This displacement was continued until the peptide was unbound (beyond the cutoff distance for nonbonded interactions). This procedure defined the dissociation pathway of the peptide through the primary and secondary cavities.

US simulations were performed using the AMBER16 software package^23^ to determine the potential of mean force (PMF) along the dissociation pathway. In the US approach, a series of windows were distributed evenly along the reaction coordinate, with conformational sampling within each window facilitated by applying an external biasing potential for 10 ns. Sampling in adjacent windows exhibited overlap, enabling reconstruction of the unbiased PMF by subsequently removing the biasing potentials. Compared with the previously reported simulations, the size of the periodic simulation box was significantly increased to prevent potential artifacts in PMF calculations that could arise from an insufficient box size during guest molecule movement. Upon completion of all US molecular dynamics simulations, data collected from the various windows were integrated along the reaction coordinate to compute the PMF profile for the entire structural transition process using the Weighted Histogram Analysis Method (WHAM)^24^ with the implementation provided by Alan Grossfield.^25^ For each complex, two simulations were performed, in total, 18 simulations were performed. In the WHAM analysis, the iteration tolerance was set to 0.001, and the temperature was maintained at 300 K.

## 3 Results and Discussion

### 3.1 ITC Analysis of Cyclodextrin–KR12-Lipopeptide Binding Interactions

The ITC technique has been successfully applied to gain some insight into the binding interactions of cyclodextrins with KR12-lipopeptides. Representative ITC binding isotherms are presented in Figures 1 and 2, while the corresponding thermodynamic parameters are summarized in Table 1. In the case of γ-CD complexes, the observed heat effects were too small to provide reliable data. This phenomenon can likely be attributed to the weaker binding interactions in this case resulting from the wider hydrophobic cavity of γ-CD. This affects the stability of the resulting complexes. Therefore, only α-CD and β-CD inclusion complexes were analyzed using a model assuming one set of binding sites. The model provided the best fit between the calculated and experimental data.

**Figure 1:**
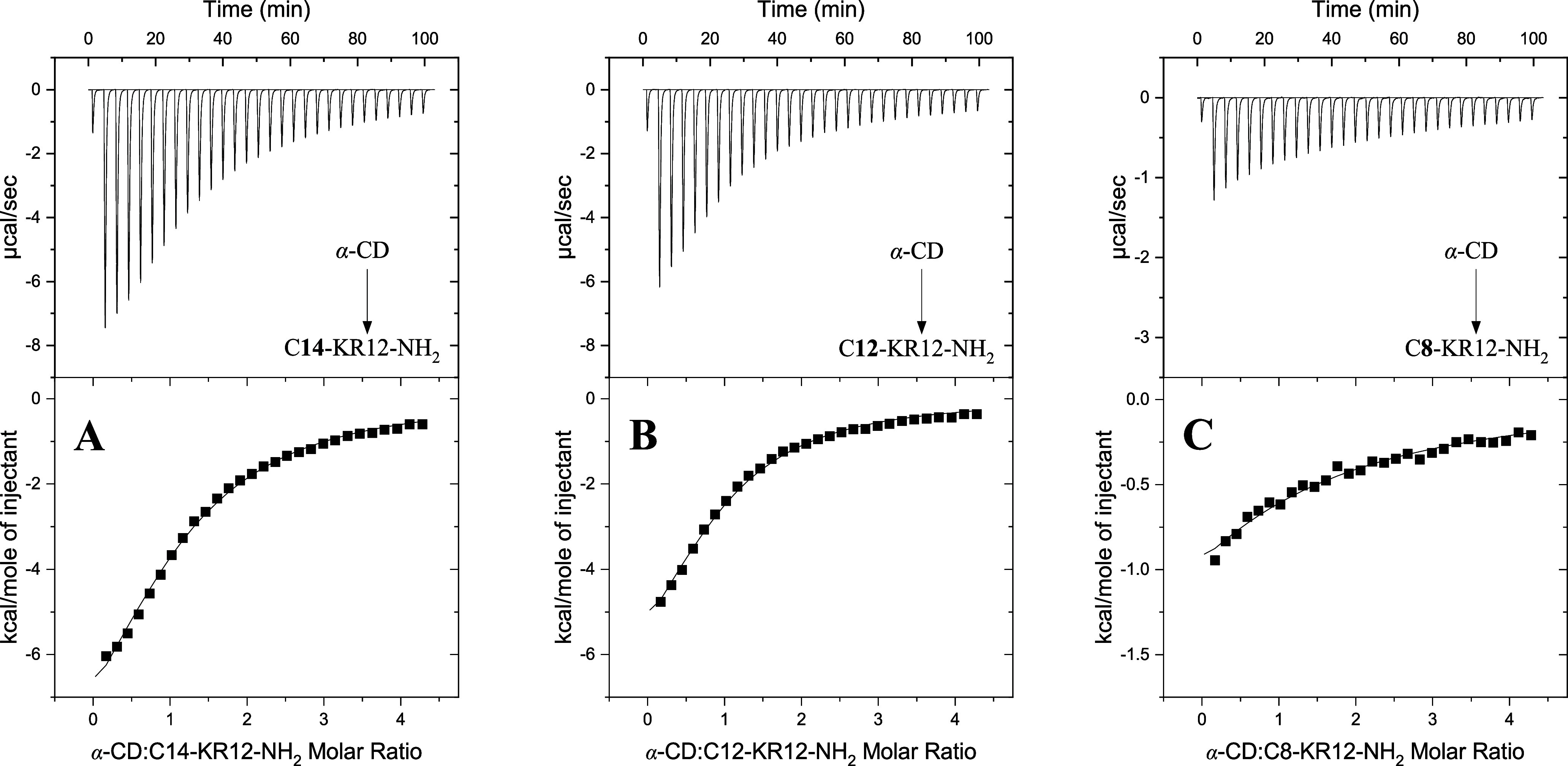
Calorimetric titration isotherms of binding interactions of the lipopeptides with α-CD in water at 298.15 K.

**Figure 2:**
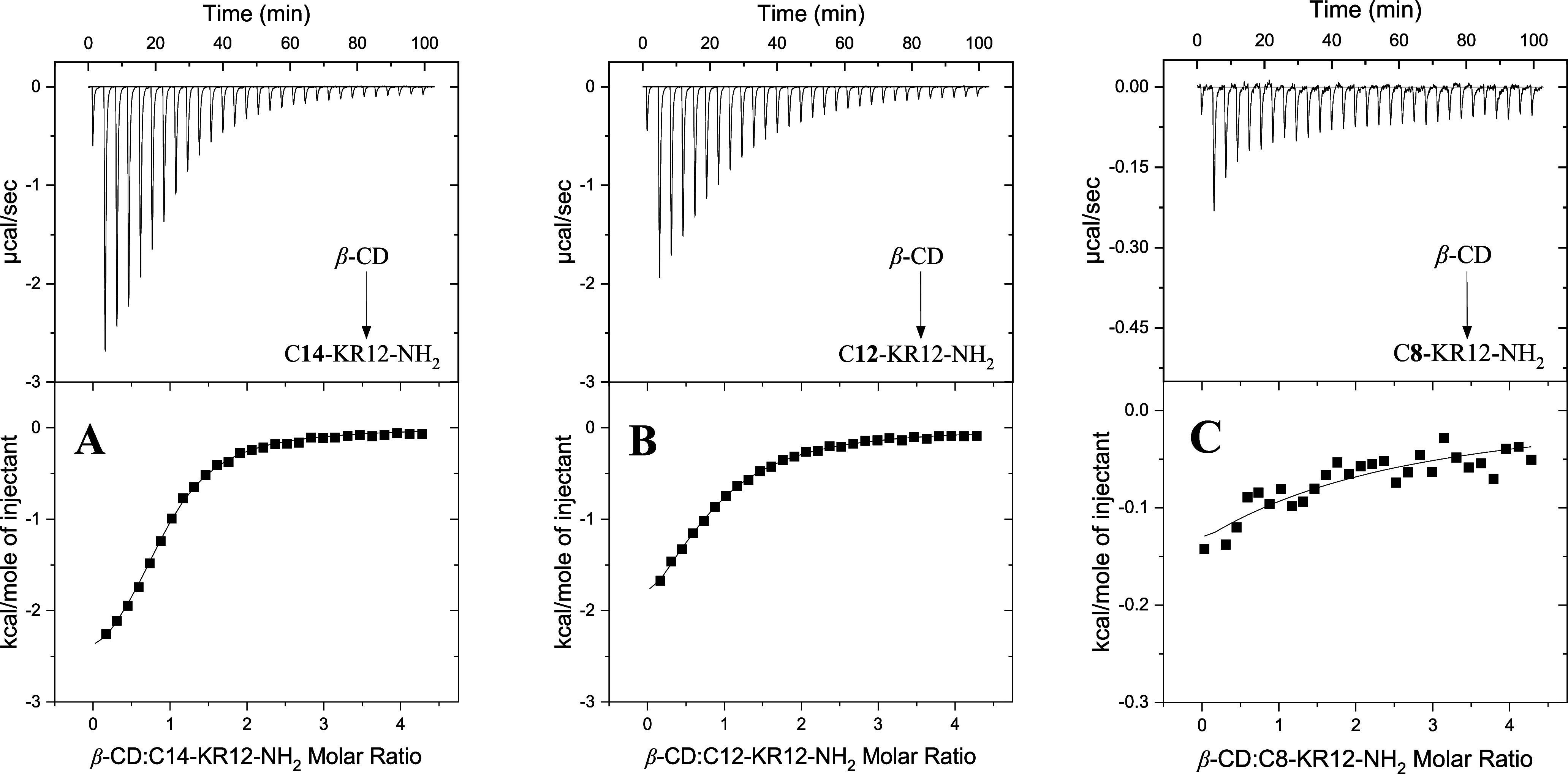
Calorimetric titration isotherms of binding interactions of the lipopeptides with β-CD in water at 298.15 K.

**Table 1:** Thermodynamic Parameters of Binding Interactions of the Lipopeptides with α-CD and β-CD in Water at 298.15 K (Standard Deviation Values in Parentheses)

parameter	C14-KR12-NH_2_	C12-KR12-NH_2_	C8-KR12-NH_2_
ITC data for α-CD-(KR12-lipopeptide) complexes			
*N*	1.10 (±0.05)	0.89 (±0.01)	0.92 (±0.01)
log*K*_(ITC)_	3.78 (±0.03)	3.87 (±0.02)	3.19 (±0.03)
Δ*G*_(ITC)_ [kcal mol^–1^]	–5.16 (±0.04)	–5.28 (±0.02)	–4.36 (±0.04)
Δ*H*_(ITC)_ [kcal mol^–1^]	–11.46 (±0.63)	–8.68 (±0.12)	–4.11 (±0.14)
*T*Δ*S*_(ITC)_ [kcal mol^–1^]	–6.30	–3.40	0.25
ITC data for β-CD-(KR12-lipopeptide) complexes			
*N*	0.89 (±0.01)	0.77 (±0.02)	0.75 (±0.03)
log*K*_(ITC)_	4.38 (±0.02)	3.97 (±0.02)	3.16 (±0.11)
Δ*G*_(ITC)_ [kcal mol^–1^]	–5.98 (±0.03)	–5.42 (±0.02)	–4.30 (±0.15)
Δ*H*_(ITC)_ [kcal mol^–1^]	–2.93 (±0.05)	–3.01 (±0.10)	–0.80 (±0.12)
*T*Δ*S*_(ITC)_ [kcal mol^–1^]	3.05	2.41	3.50

### 3.2 Stoichiometry and the Strength of the Interactions

ITC measurements revealed that α-CD and β-CD form complexes with 1:1 host-to-guest stoichiometry (Table 1). The length of the hydrocarbon chain affects the strength of the interactions, with longer lipopeptide chains forming more thermodynamically stable complexes with both α-CD and β-CD. The C14-KR12-NH_2_ peptide exhibited a higher affinity for β-CD than for α-CD. This behavior is probably since the longer hydrophobic tail (C14-) fitting more easily into the larger β-CD cavity, thereby strengthening the hydrophobic interactions. In contrast, the two other lipopeptides, octanoylated (C8-) and laurylated (C12-) KR12-lipopeptides, showed no notable differences in the binding affinity between these two cyclodextrins.

### 3.3 Dependence of the Length of the Chain of Fatty Acid on Cyclodextrin Interactions

The observed strengthening of binding interactions as the hydrocarbon chain length increases is consistent with previous studies that have emphasized the importance of chain length as a key structural feature of low-molecular-weight ligands, such as fatty acids and surfactants, in promoting hydrophobic interactions with the cyclodextrin cavity.^10,11,26,27^ It is important to note that for ligands with shorter hydrophobic tails, electrostatic interactions between the charged headgroup and the cyclodextrin rim serve as an additional factor—besides hydrophobic interactions—in enhancing the stability of the resulting inclusion complexes. This phenomenon has previously been observed in guest–host complexes formed by β-cyclodextrin and a series of alkyl sulfates with hydrophobic chains of different lengths, namely, sodium dodecyl sulfate (C_12_–OSO_3_Na), sodium decyl sulfate (C_10_–OSO_3_Na), and sodium octyl sulfate (C_8_–OSO_3_Na).^10^ However, these interactions play a less significant role compared with the penetration of the hydrophobic tail into the cyclodextrin cavity, which is the primary driver of complex formation.

### 3.4 The Thermodynamic Parameters of the Interactions

The nature of the binding interactions reflects itself in the thermodynamic parameters related to the formation of the complexes under study (Table 1). The binding of lipopeptides to α-cyclodextrin (α-CD) is primarily enthalpy-driven (|Δ*H*| > |*T*Δ*S*|). This is particularly pronounced for lipopeptides with longer alkyl chains as a more extensive hydrophobic contact surface strengthens the noncovalent interactions—primarily van der Waals forces and hydrogen bonds. The resulting tight fit guest molecule to the host facilitates favorable electrostatic interactions between the positively charged lipopeptide moiety and the cyclodextrin rim. However, this tight encapsulation also restricts the conformations of the lipopeptide, resulting in a loss of molecular flexibility and an entropic penalty (*T*Δ*S* < 0). The overall binding is therefore stabilized because the large, favorable enthalpy gain significantly outweighs this unfavorable entropic contribution. By contrast, interactions with β-cyclodextrin (β-CD) demonstrate a different thermodynamic balance. Binding is significantly less exothermic, and entropy plays a dominant role in driving complexation (*T*Δ*S* > 0). This is especially evident for lipopeptides with shorter hydrophobic chains (e.g., C8-KR12-NH_2_). The larger β-CD cavity provides a better fit for the hydrophobic guest, enabling the displacement of ordered water molecules from both the cavity and the lipopeptide surface into the bulk solvent. This release of structured water generates a substantial, favorable entropy change, which becomes the primary contributor to the binding free energy (Δ*G*). While moderate enthalpic stabilization from noncovalent interactions still occurs, the overall thermodynamic signature shifts from enthalpic to entropic dominance. Thus, the ITC data revealed that the size of the cyclodextrin cavity is the key factor in determining the thermodynamic profile: α-CD binding is dominated by enthalpy, whereas β-CD binding is more entropy-driven. This distinction highlights the importance of host–guest complementarity in determining the fundamental forces that govern these supramolecular interactions.

### 3.5 The Effect of Inclusion Complex Formations on the Secondary Structure of Lipopeptides

The ability to form stable host–guest complexes allows for the alteration of key peptide properties, such as enhancing the solubility of poorly soluble compounds, improving their stability and bioavailability, or modulating their biological activity, for instance, reducing toxicity. For the studied lipopeptides, which belong to the class of AMPs capable of disrupting bacterial cell membranes and causing lysis,^16^ the formation of inclusion complexes with cyclodextrins is particularly significant. The α-helical secondary structure of AMPs is essential to their biological activity. It affects their ability to not only interact with membranes but can also enhance their antimicrobial effectiveness. The formation of inclusion complexes can alter the conformation of the peptide chain and, as a result, impact the biological activity of lipopeptides. To ensure valid and comparable results, CD measurements were performed under conditions identical to those previously established in the literature^7^—specifically, 50 mM cacodylate buffer at pH 5. The choice of buffer is critical here as previous studies under these conditions have demonstrated a direct and measurable effect on the lipopeptide conformation. These studies showed that C12-KR12-NH_2_ and C14-KR12-NH_2_ adopt α-helical contents of 36% and 49%, respectively.^7^ In contrast, the C8-KR12-NH_2_ peptide does not display an α-helical structure under the same conditions. It can therefore be assumed that lipopeptides with longer hydrocarbon chains (e.g., C14-KR12-NH_2_) exhibit enhanced interactions of the fatty acid moiety with the hydrophobic residues of the KR12 peptide, contributing to stabilization of the peptide backbone and promoting the adoption of a more ordered, α-helical conformation. Given the well-documented sensitivity of peptide secondary structure to environmental factors such as buffer composition, ionic strength, and pH, the consistent use of this standardized buffer system allows for the direct comparison of results and isolates the impact of cyclodextrin complexation on peptide conformation from other experimental variables. Based on the CD spectra (Figure 3), it has been found that the formation of inclusion complexes with cyclodextrins disrupts these interactions by trapping the chain of fatty acid residues inside the cyclodextrin cavity. As a result, the initial α-helical content of C14-KR12-NH_2_ decreases from 49% to 28% when forming a complex with α-CD, and to 34% with β-CD (Figure 3). This effect is even more pronounced in the interaction between the C12-KR12-NH_2_ peptide and cyclodextrins, where the formation of inclusion complexes leads to a complete loss of the α-helical conformation.

**Figure 3:**
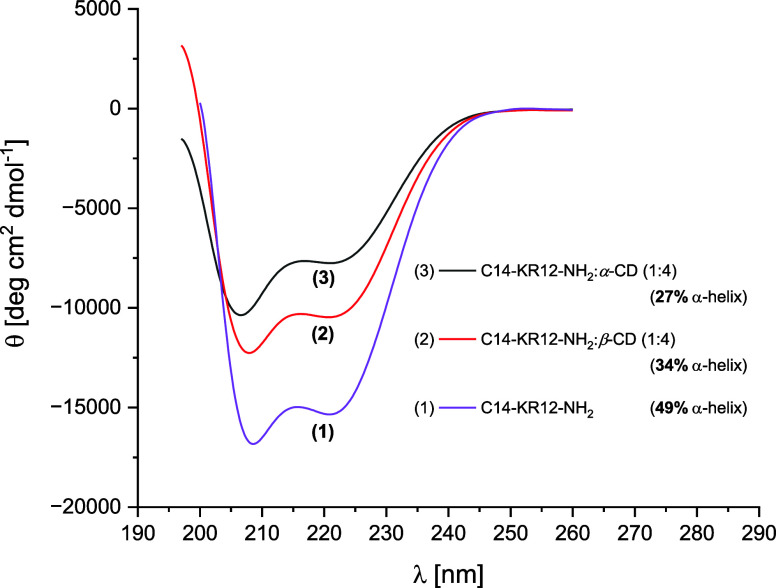
CD spectra of C14-KR12-NH_2_ and its α-CD and β-CD inclusion complexes in 50 mM cacodylate buffer of pH 5, at 298.15 K.

### 3.6 Molecular Dynamics Simulations—Atomistic Insights into the Nature of the Interactions

Molecular dynamics (MD) simulations were employed to investigate the molecular mechanism underlying the formation of inclusion complexes between lipopeptides and cyclodextrins, focusing on elucidating the structural, dynamic, and thermodynamic factors that influence these interactions. Particular attention was given to two key aspects: the entry pathway of the guest molecule into the cyclodextrin cavity—considering both the primary and secondary entrances—and the influence of structural features such as the hydrocarbon chain length of the lipopeptide and the cavity size of the cyclodextrin (α-, β-, or γ-cyclodextrin) on the stability of the formed host–guest complexes.

The mechanisms by which a guest molecule enters the cyclodextrin cavity are affected by the mutual affinity of the reactants involved in the reaction. These interactions are noncovalent, involving hydrogen bonds, electrostatic interactions, and van der Waals interactions, which facilitate the guest to be incorporated and bind reversibly within the host structure.

Generally, a guest molecule can enter a cyclodextrin cavity via four possible pathways: through the head of the molecule into either the primary or secondary cavity or through the tail of the molecule into one of these cavities. Additionally, it is essential to consider that an equilibrium may exist among various competing pathways through which a guest can enter the host’s cavity. It has previously been reported for 1-alkylsulfates and 1-alkylsulfonates that the specific mechanisms of guest entry are influenced by factors such as the length of the hydrophobic tails, the size of the polar headgroup, and the charge distribution on the headgroups.^10,11,28^ These structural features are essential for molecular recognition, allowing for the selective and reversible encapsulation of guests.

The present investigations rely exclusively on MD simulation data without the application of initial molecular docking procedures. At the outset of each simulation, the guest molecule (the lipopeptide) was positioned within the cavity of each cyclodextrin variant. Subsequently, a visual analysis of the resulting trajectories was conducted.

In all cases, the lipopeptides entered the cyclodextrin cavity through the hydrocarbon chain. This contrasts with the behavior observed for 1-alkylsulfates and 1-alkylsulfonates, for which entry pathways via the polar head of the guest molecule into either the primary or secondary cavity were also possible. Thus, for each lipopeptide–cyclodextrin complex, only two possible entry pathways were analyzed, namely, via the primary (Complex 1) and the secondary (Complex 2) cyclodextrin cavities (Figure 4).

**Figure 4:**
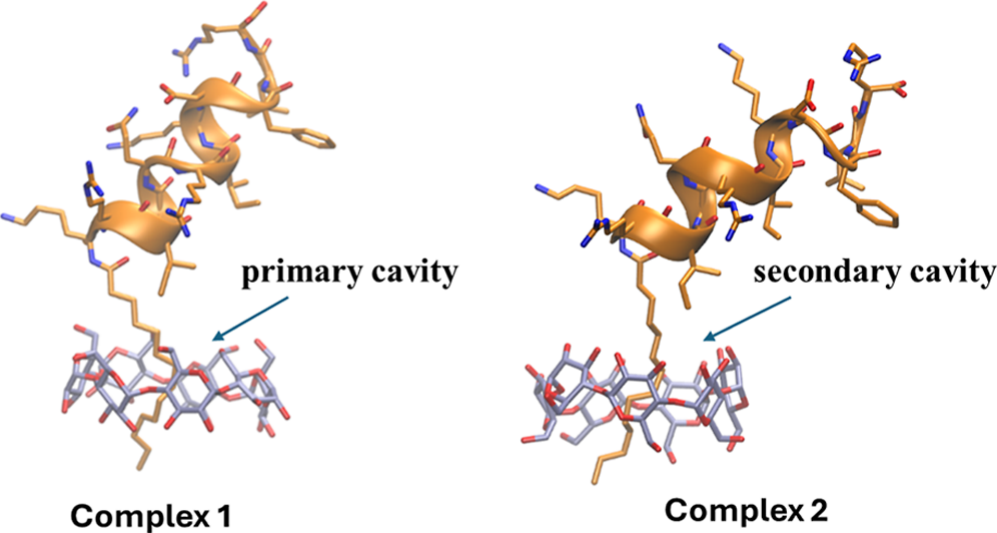
Two representative potential entrance pathways for the lipopeptide (C14-KR12-NH_2_) into β-cyclodextrin cavity: complex 1 (the lipopeptide enters via the primary cavity), complex 2 (the lipopeptide enters via the secondary cavity).

The MM-GBSA and LIE binding free-energy calculations showed that both electrostatic and van der Waals interactions contribute more to the stability of the complex when the lipopeptide is inserted through the wider (secondary) more hydrophilic side of the host molecule (complex 2). This trend is observed regardless of the size of the cyclodextrin cavity but is more noticeable in the case of peptide conjugates with longer hydrocarbon chains (Tables S1 and S2). On the other hand, it is important to note that in most investigated cases, the estimated free energy values for both primary/secondary host–guest conformations fall within the range of calculation variance (Figure 5). As a result, a chemical equilibrium between the two possible conformers, namely, complex 1 ⇌ complex 2, is likely to exist.

**Figure 5:**
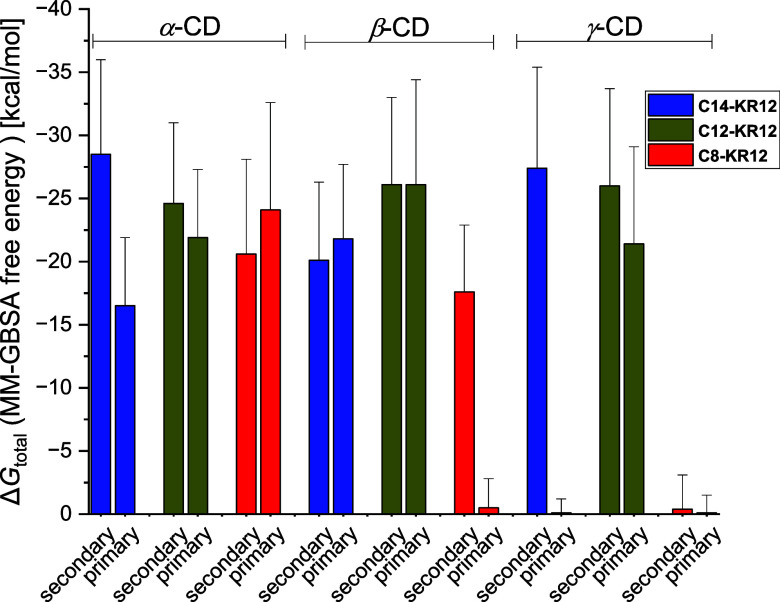
Relative stability of two potential KR12-lipopeptide/cyclodextrin conformers (complex 1 versus complex 2) through MM-GBSA free-energy calculations.

Theoretical findings align with experimental data, indicating that the cavity size of the cyclodextrin molecule directly influences the stability of the formed inclusion complexes. Specifically, γ-cyclodextrin, owing to its larger internal volume, forms markedly less stable complexes with C8-KR12-NH_2_ compared to α-cyclodextrin. This suggests that excessive cavity size may compromise optimal host–guest interactions for shorter hydrocarbon chains. In contrast, for C12-KR12-NH_2_ and C14-KR12-NH_2_ complexes, the inclusion stability appears to be comparable across all cyclodextrin types, indicating that longer lipopeptide chains may better accommodate varying cavity sizes. These findings collectively suggest that longer hydrocarbon chains enhance complex stability, likely due to improved hydrophobic encapsulation.

To provide additional information about the nature of the binding interactions, US simulations were employed. The binding free-energy dissociation profile was determined, including the free-energy barrier (Table S3). However, no significant differences were observed in the calculated values for the investigated primary and secondary cavity host–guest complexes except for the C14-KR12-NH_2_/α-CD complexes, where complex 2 exhibits notably higher stability. Both electrostatic and van der Waals interactions appear to contribute comparably to the stabilization of the complexes. In addition to these contributions, hydrogen bonding plays a significant role in maintaining the structural integrity of the host–guest assemblies by providing directional, yet rather nonspecific, interactions (Table S4). While hydrogen bonds contribute to local stabilization and help to position the lipopeptide within the cyclodextrin cavity, they do not confer high specificity in the binding process. Thus, the overall stability arises from a synergistic balance of hydrophobic effects, electrostatic forces, van der Waals contacts, and nonspecific hydrogen bonds.

## 4 Conclusions

This study provides the first comprehensive characterization of the interactions among α-, β-, and γ-cyclodextrins and a series of KR12-derived lipopeptides. Employing a model system approach, we elucidated the fundamental physicochemical principles governing lipopeptide–cyclodextrin inclusion complex formation and its consequential impact on the lipopeptide secondary structure. Our results establish that the stability of a complex is determined by the precise structural match between the length of the peptide’s hydrophobic chain and the size of the cyclodextrin cavity. The thermodynamics of complexation were found to vary significantly: α-CD binding is primarily enthalpy-driven, facilitated by strong hydrogen bonding and van der Waals forces, whereas β-CD complexation with shorter chains tends to be less exothermic and more entropy-driven. A key structural finding is that encapsulation of the lipopeptide’s fatty acid chain within the cyclodextrin cavity disrupts its native α-helical conformation, a critical determinant of antimicrobial activity. Molecular dynamics simulations confirmed a dynamic equilibrium with the hydrocarbon chain able to access both the primary and secondary cavities of the cyclodextrin.

The results obtained suggest that cyclodextrins act as competitive ligands by complexing the lipid tail of the lipopeptide. Consequently, this phenomenon may lead to the inhibition or hindrance of the essential hydrophobic anchoring step required for bacterial membrane disruption. This mechanism can effectively weaken the antimicrobial function of lipopeptide, highlighting the direct pathway of modulation of its activity in the presence of cyclodextrin. The ability of cyclodextrins to shield hydrophobic moieties provides a powerful means of mitigating the nonselective cytotoxicity of lipopeptides toward mammalian cells, protecting their membranes from undesired damage.

This work deepens our understanding of the structure–activity relationships involved, opening avenues for the design of safer antimicrobial therapeutics. To translate the presented findings into applications, future research must exploit cyclodextrin complexation as a tunable design principle. This requires a detailed investigation into how these inclusion complexes interact with physiologically relevant lipid environments, accounting for factors like membrane composition, to deliberately program membrane selectivity. This approach provides a clear pathway for engineering safer and more effective lipopeptide prodrugs and formulations.

## Supplementary Material



## Notes

MM-GBSA free-energy calculations; LIE free-energy calculations; PMF analysis; and hydrogen-bond analysis for α-, β-, and γ-cyclodextrin–(KR12-lipopeptide) complexes (PDF)
