# Supplementary material for: Cyclodextrin and KR12-Lipopeptide Interactions: A Thermodynamic View of Binding Mechanisms and Impact on the Structure of α‑Helical Peptides
Source: J Phys Chem B. 2026 Jan 19;130(4):1167–74. doi: 10.1021/acs.jpcb.5c06749 (PMC13296729; doi:10.1021/acs.jpcb.5c06749)
Supplement: Supplementary file 1 [file jp5c06749_si_001.pdf]

Supporting information to:

Cyclodextrin and KR12-Lipopeptide Interactions: A Thermodynamic View of Binding Mechanisms and Impact on the Structure of α-Helical Peptides

Martyna Kapica[1], Ola Grabowska[1], Elżbieta Kamysz[1], Julia Kamysz[2], Sergey A. Samsonov[1]*, Dariusz Wyrzykowski[1]

[1]Faculty of Chemistry, University of Gdańsk, Wita Stwosza 63, 80-308 Gdańsk, Poland

[2]Inter-faculty Individual Studies in Mathematics and Natural Sciences, University of Warsaw, Stefana Banacha 2C, 02-097 Warsaw, Poland

* Correspondence: sergey.samsonov@ug.edu.pl (S.A.S.)

Table S1. MM-GBSA free energy calculations for *α*-, *β*-, and *γ*-cyclodextrin–(KR12 Lipopeptide) complexes.

*MM-GBSA free energy calculations for **α-CD-(KR12 lipopeptide)** complexes*			
Complex	ΔG_{total}, kcal/mol	ΔG_{el}, kcal/mol	ΔG_{vdW}, kcal/mol
Side2_C14	-28.5 ± 7.5	-33.1± 20.5	-44.6 ± 8.6
Side1_C14	-16,5 ± 5.4	-9,1± 14.3	-28.5± 7.5
Side2_C12	-24.6± 6.4	-26.4± 18.6	-37.1± 6.5
Side1_C12	-21.9± 5.4	-15.4± 14.8	-34.1± 6.7
Side2_C8	-20.6± 7.5	-27.5± 18.6	-32.1± 7.4
Side1_C8	-24.1± 8.5	-22.4± 20.0	-35.0± 8.4
*MM-GBSA free energy calculations for **β-CD-(KR12 lipopeptide)** complexes*			
Complex	ΔG_{total}, kcal/mol	ΔG_{el}, kcal/mol	ΔG_{vdW}, kcal/mol
Side2_C14	-20.1±6.2	-18.9±15.9	-38.9±6.2
Side1_C14	-21.8±5.9	-15.3±15.4	-35.6±6.3
Side2_C12	-26.1±6.9	-28.0±14.9	-42.3± 6.5
Side1_C12	-26.1±8.3	-37.2±29.9	-41.7±10.5
Side2_C8	-17.6±5.3	-26.3±21.0	-34.5±5.4
Side1_C8	-0.5±2.3	-0.9±5.0	-1.1±4.5
*MM-GBSA free energy calculations fo **γ-CD-(KR12 lipopeptide)** complexes*			
Complex	ΔG_{total}, kcal/mol	ΔG_{el}, kcal/mol	ΔG_{vdW}, kcal/mol
Side2_C14	-27.4 ±8.0	-31.3 ±18.8	-45.5 ±8.9
Side1_C14	-0.1 ±1.1	-0.1 ±1.2	-0.3 ±2.6
Side2_C12	-26.0 ±7.7	-42.0 ±21.7	-44.9 ± 9.4
Side1_C12	-21.4 ±7.7	-24.2 ± 17.7	-34.0 ±8.4
Side2_C8	-0.4 ±2.7	-0.7 ±4.8	-0.8 ±4.4
Side1_C8	-0.1 ±1.4	-0.8 ±5.8	-0.4 ±2.5

ΔG_{total}, ΔG_{el}, ΔG_{vdW} are total, *in vacuo* electrostatics and van der Waals components, respectively.

Table S2. LIE free energy calculations for *α*-, *β*-, and *γ*-cyclodextrin–(KR12 Lipopeptide) complexes.

*LIE free energy calculations for **α-CD-(KR12 lipopeptide)** complexes*	
Complex	Δ*G*, kcal/mol
Side2_C14	-44.7 ±8.7
Side1_C14	-28.3 ±7.4
Side2_C12	-37.1 ±6.6
Side1_C12	-34.1±6.7
Side2_C8	-32.1 ±7.5
Side1_C8	-35.0 ±8.6
*LIE free energy calculations for **β-CD-(KR12 lipopeptide)** complexes*	
Complex	Δ*G*, kcal/mol
Side2_C14	-38.6 ±6.2
Side1_C14	-34.5±6.0
Side2_C12	-42.2±6.6
Side1_C12	-41.8±10.8
Side2_C8	-34.4±5.4
Side1_C8	-1.0±4.5
*LIE free energy calculations for **γ-CD-(KR12 lipopeptide)** complexes*	
Complex	Δ*G*, kcal/mol
Side2_C14	-45.5 ± 9.1
Side1_C14	-0.2 ±2.6
Side2_C12	-45.0±9.5
Side1_C12	-34.0±8.5
Side2_C8	-0.8 ±4.4
Side1_C8	-0.4 ±2.5

Table S3. PMF analysis for *α*-, *β*-, and *γ*-cyclodextrin–(KR12 Lipopeptide) complexes.

PMF analysis *for **α-CD-(KR12 lipopeptide)** complexes*		
Complex	ΔG_{bind}, kcal/mol	$\Delta G_{barrier}$, kcal/mol
Side2_C14	-4.14	6.26
Side1_C14	-0.005	0.24
Side2_C12	-1.08	1.87
Side1_C12	-1.30	2.07
Side2_C8	-1.15	1.61
Side1_C8	-0.80	1.88
PMF analysis *for **β-CD-(KR12 lipopeptide)** complexes*		
Complex	ΔG_{bind}, kcal/mol	$\Delta G_{barrier}$, kcal/mol
Side2_C14	-0.72	1.44
Side1_C14	-0.78	1.63
Side2_C12	-0.54	1.03
Side1_C12	-0.27	1.43
Side2_C8	-0.99	1.98
Side1_C8	-0.003	0.27
PMF analysis *for **γ-CD-(KR12 lipopeptide)** complexes*		
Complex	ΔG_{bind}, kcal/mol	$\Delta G_{barrier}$, kcal/mol
Side2_C14	-0.12	0.47
Side1_C14	-1.64	1.97
Side2_C12	-0.54	0.91
Side1_C12	-0.04	0.74
Side2_C8	-0.82	1.08
Side1_C8	-0.41	0.83

ΔG_{bind} and $\Delta G_{barrier}$ are the binding free energy and free energy barrier, respectively.

Table S4. Hydrogen Bond Analysis for *α-, β-,* and *γ*-cyclodextrin–(KR12 Lipopeptide) complexes.

*Hydrogen bond analysis for **α-CD-(KR12 lipopeptide)** complexes*			
**Complex**	**Acceptor atom**	**Donor heavy atom**	**Occupancy**
Side2_C14	VAL_5@O	4Glc_6@O3	0.17
	VAL_5@O	4Glc_6@O2	0.14
	GLN_6@O	4Glc_1@O3	0.14
	GLN_6@OE1	4Glc_2@O2	0.10
Side1_C14	-	-	-
Side2_C12	C12_1@O14	4Glc_5@O2	0.10
Side1_C12	-	-	-
Side2_C8	VAL_5@O	4Glc_4@O2	0.12
	ASP_10@OD1	4Glc_5@O2	0.10
Side1_C8	-	-	-
*Hydrogen bond analysis for **β-CD-(KR12 lipopeptide)** complexes*			
**Complex**	**Acceptor atom**	**Donor heavy atom**	**Occupancy**
Side2_C14	-	-	-
Side1_C14	-	-	-
Side2_C12	ILE_4@O	4Glc_1@O2	0.12
Side1_C12	GLN_6@O	4Glc_3@O2	0.11
Side2_C8	-	-	-
Side1_C8	-	-	-
*Hydrogen bond analysis for **γ-CD-(KR12 lipopeptide)** complexes*			
**Complex**	**Acceptor atom**	**Donor heavy atom**	**Occupancy**
Side2_C14	GLN_14@O	4Glc_3@O2	0.15
	GLN_14@O	4Glc_3@O3	0.13
Side1_C14	-	-	-
Side2_C12	LYS_2@O	4Glc_3@O3	0.19
	GLN_6@O	4Glc_2@O2	0.18
	C12_1@O14	4Glc_7@O3	0.13
	PHE_11@O	4Glc_3@O2	0.10
	LYS_2@O	4Glc_3@O2	0.10
Side1_C12	-	-	-
Side2_C8	-	-	-
Side1_C8	-	-	-

Hydrogen bonds with the occupancy higher than 0.1 are listed. Atom names correspond to the AMBER nomenclature.
